# College and Hospital Notes

**Published:** 1899-08

**Authors:** 


### COLLEGE AND HOSPITAL NOTES.

---

THE Buffalo Fresh Air Mission Hospital at Athol Springs opened July 1, 1899. It is situated on the lake shore, ten miles from Buffalo, and is reached in twenty-five minutes by train. The hospital is thus most favorably placed for the treatment of infants and children suffering from cholera infantum and other diarrheal diseases. Physicians are urged to send to the hospital any case of cholera infantum or infantile diarrhea which would be benefited by the country air. The attention of the medical profession is also called to the fact that until the wards are filled with cholera infantum cases, children afflicted with any medical or surgical disease, not contagious, will be gladly received. The hospital is free, is equipped with all necessary apparatus, and is provided with resident physicians and trained nurses. Information furnished by Dr. Irving M. Snow, 476 Franklin street; Dr. DeWitt H. Sherman, 666 Main street, or at the Fitch Hospital, 165 Swan street. In urgent cases a telephone message can be sent to the Fitch Institute.

---

THE Hospital College of Medicine, the medical department of Central University of Kentucky, held its annual commencement at Louisville, June 30, 1899.

The announcement of the college was made by Dr. L. S. McMurtry, president of the medical faculty. Dr. McMurtry stated that last year the number of graduates was 130, while this year there were only 18. He explained that this was due to the increasing of the term from three to four years.

The presentation of candidates for the degree of doctor of medicine was made by Dr. Taylor, and he also awarded the honors and prizes. The Rev. Dr. Blanton, chancellor, conferred the degrees and Col. Bennett H. Young delivered the address to the graduates. He spoke in the highest terms of the graduates and paid a high tribute to Louisville and Kentucky colleges, particularly to Central University. The exercises closed with a benediction by Rev. Dr. Hemphill.

---

A HOSPITAL for sailors afflicted with tuberculosis has been established on the old Fort Stanton military reservation in New Mexico. This has been done at the instance of surgeon-general Walter Wyman, marine hospital service.

Some years ago, when Dr. Bratton of the marine hospital corps became affected with consumption and was obliged to seek his health in New Mexico, Surgeon-general Wyman instructed him to report in great detail concerning various sites in the territory that might be used for sanatorium sites. Ever since the report was submitted the surgeon-general has been endeavoring to interest the authorities in the matter, with the result already noted. The Fort Stanton reservation is one of the finest available for the use to which it will now be devoted. It consists of more than 10,000 acres, situated in the Capitan Mountains, 6,000 feet above the sea. As soon as convenient all the consumptive patients who are now in the various marine hospitals of the country will be moved to the reservation. As Surgeon-general Wyman says, this movement is to isolate consumptives in a sanatorium, located in a country affording especial climatic advantages, is in line with the movement which is now going on all over the world to suppress the dread disease.

---

THE Buffalo state pathological laboratory has attracted the attention of the British parliament. According to a recent associated press dispatch, in reply to Sir Charles Cameron, liberal member for the Bridgeton division of Glasgow in the House of Commons, July 11th, 1899, the parliamentary secretary of the foreign office, William St. John Broderick, said the attention of the foreign office had not been previously called to the fact that owing to the death from cancer the New York legislature had endowed a laboratory at Buffalo to study the disease. But the under secretary added the British Charge d'Affaires at Washington would forthwith be asked to furnish the government with all possible information regarding the institution.

